# Neuronal precursor cell proliferation in the hippocampus after transient cerebral ischemia: a comparative study of two rat strains using stereological tools

**DOI:** 10.1186/2040-7378-2-8

**Published:** 2010-04-06

**Authors:** Jesper Kelsen, Marianne H Larsen, Jens Christian Sørensen, Arne Møller, Jørgen Frøkiær, Søren Nielsen, Jens R Nyengaard, Jens D Mikkelsen, Lars Christian B Rønn

**Affiliations:** 1 The Water and Salt Research Centre, University of Aarhus, DK-8000 Aarhus C, Denmark; 2 Institute of Clinical Medicine, University Hospital of Aarhus, Brendstrupgaardsvej 100, DK-8200 Aarhus N, Denmark; 3 Department of Neurosurgery, University Hospital of Copenhagen (Rigshospitalet), Blegdamsvej 9, DK-2100 Copenhagen Ø, Denmark; 4 Department of Neuroscience and Pharmacology, The Panum Institute, University of Copenhagen, Blegdamsvej 3, DK-2200 Copenhagen N, Denmark; 5 Department of Neurosurgery, University Hospital of Aarhus, Nørrebrogade 44, DK-8000 Aarhus C, Denmark; 6 PET-centre, University Hospital of Aarhus, Nørrebrogade 44, DK-8000 Aarhus C, Denmark; 7 Department of Cell Biology, Institute of Anatomy, Building 1230, University of Aarhus, DK-8000 Aarhus C, Denmark; 8 Stereology and Electron Microscopy Research Laboratory and MIND Center, Danish Neuroscience Centre, University Hospital of Aarhus, Nørrebrogade 44, DK-8000 Aarhus C, Denmark; 9 Neurobiology Research Unit, University Hospital of Copenhagen (Rigshospitalet), Building 9201, Blegdamsvej 9, DK-2100 Copenhagen Ø, Denmark; 10 NEUROSEARCH A/S, Pederstrupvej 93, DK-2750 Ballerup, Denmark

## Abstract

**Background:**

We are currently investigating microglial activation and neuronal precursor cell (NPC) proliferation after transient middle cerebral artery occlusion (tMCAo) in rats. This study aimed: (1) to investigate differences in hippocampal NPC proliferation in outbred male spontaneously hypertensive rats (SHRs) and Sprague-Dawley rats (SDs) one week after tMCAo; (2) to present the practical use of the optical fractionator and 2D nucleator in stereological brain tissue analyses; and (3) to report our experiences with an intraluminal tMCAo model where the occluding filament is advanced 22 mm beyond the carotid bifurcation and the common carotid artery is clamped during tMCAo.

**Methods:**

Twenty-three SDs and twenty SHRs were randomized into four groups subjected to 90 minutes tMCAo or sham. BrdU (50 mg/kg) was administered intraperitoneally twice daily on Day 4 to 7 after surgery. On Day 8 all animals were euthanized. NeuN-stained tissue sections were used for brain and infarct volume estimation with the 2D nucleator and Cavalieri principle. Brains were studied for the presence of activated microglia (ED-1) and hippocampal BrdU incorporation using the optical fractionator.

**Results:**

We found no significant difference or increase in post-ischemic NPC proliferation between the two strains. However, the response to remote ischemia may differ between SDs and SHRs. In three animals increased post-stroke NPC proliferation was associated with hippocampal ischemic injury. The mean infarct volume was 89.2 ± 76.1 mm^3 ^in SHRs and 16.9 ± 22.7 mm^3 ^in SDs (p < 0.005). Eight out of eleven SHRs had ischemic neocortical damage in contrast to only one out of 12 SDs. We observed involvement of the anterior choroidal and hypothalamic arteries in several animals from both strains and the anterior cerebral artery in two SHRs.

**Conclusions:**

We found no evidence of an early hippocampal NPC proliferation one week after tMCAo in both strains. Infarction within the anterior choroidal artery could induce hippocampal ischemia and increase NPC proliferation profoundly. NPC proliferation was not aggravated by the presence of activated microglia. Intraluminal tMCAo in SHRs gave a more reliable infarct with neocortical involvement, but affected territories supplied by the anterior cerebral, anterior choroidal and hypothalamic arteries.

## Background

The ischemic stroke accounts for the largest proportion of patients among the ischemic/hypoxic brain diseases. The neuroinflammatory response that is part of the pathobiology of ischemic stroke is presently under intense investigation. The cascade leading to activation of microglia and synthesis of cyto- and chemokines is one of the promising targets in the prevention of ischemic core enlargement [1-4]. Since the early 1980s much effort has been put into the investigation of potential neuroprotective drugs [5]. Experimental stroke research is now focusing on neuronal repair with either endogenous neuronal precursor cells (NPCs) [6] or exogenous stem cell implantation strategies [7].

Spontaneously hypertensive rats (SHRs) have been widely used in studies of transient cerebral ischemia and have several characteristics that make them suitable for these studies: (1) a higher success rate and reproducibility in transient and permanent models of middle cerebral artery occlusion [8-13]; (2) a higher basal rate of neurogenesis [14]; (3) a pronounced neuroinflammatory response after stroke [15]; and (4) hypertension is one of the most prominent risk factors in the pathogenesis of the ischemic stroke.

Inspired by the work of Marks et al. [15] and Perfilieva et al. [14], we investigated the differences in infarct distribution and NPC proliferation after tMCAo in outbred male SHRs and Sprague-Dawley rats (SDs). The aim of the present study was three-fold: (1) to investigate whether the hippocampal NPC proliferation differed between outbred male SDs and SHRs one week after tMCAo; (2) to present the practical use of the 2D nucleator combined with the Cavalieri principle and the optical fractionator in stereological tissue analysis; and (3) to report the use of an intraluminal tMCAo model in rats where the occluding filament is advanced 22 mm beyond the carotid bifurcation and the common carotid artery is clamped during tMCAo.

## Methods

We performed all experimental procedures in accordance with the Danish animal protection legislation. The experimental protocol was approved by The Danish Animal Experiments Inspectorate (license no. 2003/561-702) according to The European Community Council Directive of November 24^th ^1986 (86/609/EEC). The animals were housed in cages of two without environmental enrichment and kept in a twelve-hour light:dark cycle with free access to standard laboratory chow and water throughout the experiment.

### Study design

A total of 43 outbred male rats with a mean bodyweight of 333 ± 16 gram. The SDs were ten weeks of age, whereas the SHRs were six weeks older due to the different growth curves provided by the breeder. All animals were purchased from Taconic (Taconic, Germantown, NY 12526, USA and Taconic Europe A/S, DK-8680 Ry, Denmark), and randomized into four groups: I. SD sham (n = 9); II. SD tMCAo (n = 14); III. SHR sham (n = 7); and IV. SHR tMCAo (n = 13).

Exclusion criteria in the present study were: (1) bad clinical condition with loss of the postural reflex; (2) damage to the internal carotid artery so that reperfusion potentially could be compromised; (3) seizures and stereotypies; (4) intracranial hemorrhage; and (5) unexpected death.

### Manufacturing of the occluding filaments

**Figure 1:**
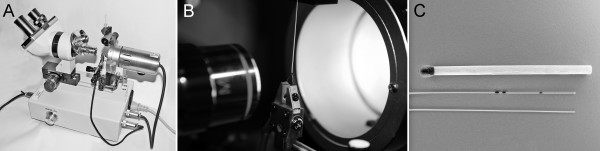
**Manufacturing of the occluding filaments**. **A **shows the MF-830 micro forge (Narishige Scientific Instrument Laboratory, Tokyo, Japan) used for the manufacturing of the occluding filaments. The tip of the microloader (Eppendorf AG, Hamburg, Germany) was positioned just above the heatable platinum wire as shown in **B**. The rounded tip was formed by increasing the temperature of the platinum wire. The heating process was activated and controlled with a foot pedal and could simultaneously be followed through the oculars of the micro forge. The microloader with the black marks has a rounded tip, whereas the microloader below has not been modified (**C**). The marks were used for the correct positioning of the microloader in the internal carotid artery. The last black mark was placed 22 mm behind the rounded tip and had to be positioned on the level of the carotid bifurcation.

We used Eppendorf microloaders (Eppendorf AG, Hamburg, Germany) and a controlled heating procedure involving a MF-830 micro forge (Narishige Scientific Instrument Laboratory, Tokyo, Japan) for the manufacture of the filaments as presented in detail in Figure 1. We defined that the tip of the occluding filaments should have a diameter between 275 and 300 μm according to Kohno et al. [16]. The diameter of all microloaders was measured at the tip, behind the tip, 1 cm and 2 cm from the tip, using CAST^® ^software (Visiopharm A/S, Hørsholm, Denmark) (Additional file 1).

### Anesthesia protocol

Anesthesia was rapidly induced by inhalation of 5% isoflurane (Baxter Isoflurane, Baxter Medical) delivered in a 65%/35% atmosphere of nitrous oxide (N_2_O) and oxygen (O_2_) at 1.0 L/min for 2 minutes. Isoflurane anesthesia was maintained throughout surgery and continuously delivered via a nose mask at a concentration of 1.2-2% in the 65%/35% N_2_O and O_2 _mixture. The left femoral artery was catheterized with a BD Neoflon™ (Becton Dickinson, Sweden) through a small incision in the left inguinal region. A PowerLab SP8 (ADInstruments, Castle Hill, NSW, Australia) was used to record mean arterial blood pressure, heart rate and rectal temperature during surgery. The first arterial blood sample was obtained within 15-20 minutes after anesthesia induction and repeated again during and after ischemia. Arterial blood parameters (pH, pCO_2 _and pO_2_) were analyzed on an ABL 500 (Radiometer, Copenhagen, Denmark) blood gas analyzer. Blood glucose and hemoglobin were determined on HemoCue Photometers (HemoCue AB, Ängelholm, Sweden).

**Figure 2:**
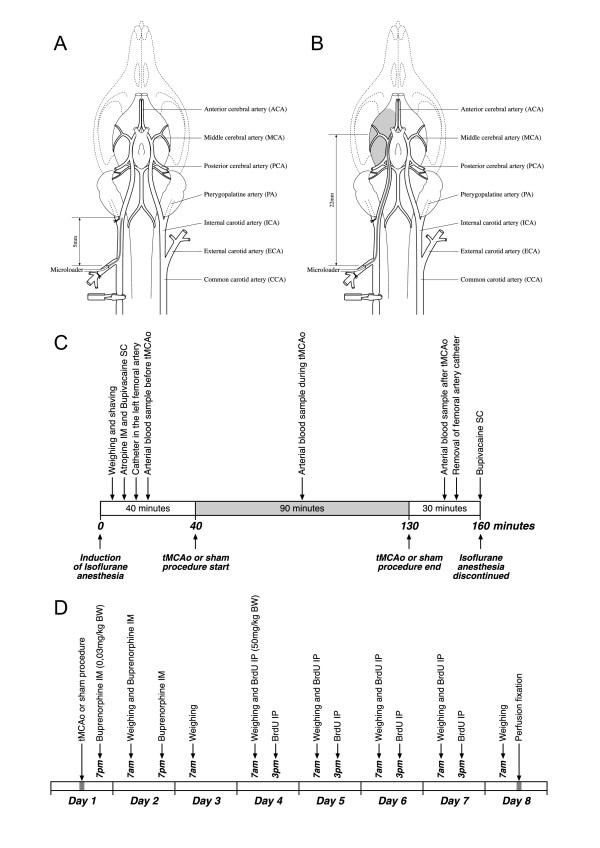
**Schematic drawings of the tMCAo model and the study schedule**. **A **and **B **are schematic drawings modified according to Longa et al. [17] of the sham and tMCAo procedure. The occluding filament (microloader) was advanced to the bifurcation of the PA and ICA in sham animals. In tMCAo rats the tip of the microloader was advanced 22 mm beyond the carotid bifurcation. Note that the PA was ligated permanently, and the ipsilateral CCA clamped temporarily during the ischemic challenge. **C **shows the time course of the surgical procedures. Each animal was on average anesthetized for 160 minutes with isoflurane, and subjected to either 90 minutes of sham surgery or tMCAo. All rats received an intramuscular injection of 50 μg/kg bodyweight atropine (Atropin SAD 1 mg/ml; Denmark) to reduce mucus production while the animals breathed spontaneously in isoflurane anesthesia. Bupivacaine (Bupivacain SAD 2.5 mg/ml; Denmark) was administered subcutaneously at the incision sites in the neck and left femoral region to avoid increase in blood pressure during surgery and to ease postoperative pain. Arterial blood gasses were obtained before, during and after surgery. **D **represents the schedule of buprenorphine (30 μg/kg BW) and BrdU (50 mg/kg BW) administration. Buprenorphine was administered as a painkiller IM twice daily within the first 48 hours after surgery. BrdU was given IP twice each day on Day 4 to Day 7. All animals were euthanized after one week. **BrdU**, 5-bromo-2'-deoxy-uridine; **BW**, bodyweight; **CCA**, common carotid artery; **ICA**, internal carotid artery; **IM**, intramuscularly; **IP**, intraperitoneally; **PA**, pterygopalatine artery; **tMCAo**, transient middle cerebral artery occlusion.

The animals recovered in clean cages without environmental enrichment and were housed two animals per cage. Buprenorphine (Temgesic^®^, 30 μg/kg bodyweight, Schering-Ploug) was administered as a painkiller twice daily for the first 48 hours after surgery. The experimental procedures are summarized in Figure 2C.

### Intraluminal transient middle cerebral artery occlusion

We used a tMCAo model modified according to Longa et al. [17] and Zarow et al. [18]. A schematic drawing of the animal model is shown in Figure 2A and 2B. The right common carotid artery was identified and isolated while surgical trauma to the adherent vagus nerve was carefully avoided. The occipital artery was permanently ligated with a 6-0 silk suture, and the superior thyroid artery was coagulated and transected to mobilize the right external carotid artery. A stump of the right external carotid artery was created by ligating the artery where it branches into the lingual and maxillary arteries. Distal to the ligature, the lingual and the maxillary artery were coagulated and cut. The right internal carotid artery and the pterygopalatine artery were mobilized, and the pterygopalatine artery was ligated permanently slightly distal to the bifurcation of the internal carotid artery and pterygopalatine artery. The rounded tip of the filament was inserted through an arteriotomy into the external carotid artery stump. The tip of the microloader was gently advanced 22 mm beyond the bifurcation of the internal carotid artery and the external carotid artery. During the 90 minutes ischemia protocol the right common carotid artery was clamped temporarily to diminish blood flow as much as possible. Rats randomized to sham surgery underwent the same regimen except that the microloader was only advanced to the bifurcation of the pterygopalatine artery and the internal carotid artery (Figure 2A).

After withdrawal of the microloader, the external carotid artery stump was ligated and the common carotid artery clamp released.

### BrdU labeling of neuronal precursor cells

5-bromo-2'-deoxy-uridine (BrdU) was administered intraperitoneally (50 mg/kg bodyweight, Sigma-Aldrich, St. Louis, MO, USA) two times daily with an eight-hour interval on Day 4 to 7 (Figure 2D).

### Brain tissue handling

The animals were deeply anesthetized with mebumal intraperitoneally (Mebumal SAD 50 mg/ml; Denmark) one week after being subjected to sham or tMCAo procedures. Transcardial brain perfusion fixation was initiated, using ice-cold isotonic saline for two minutes followed by 4% paraformaldehyde for eight minutes at a flow rate of 20 ml/min. The brains were immersed overnight in 4% paraformaldehyde at 4°C and transferred in phosphate-buffered 0.9% saline at 4°C. Three days before cryosectioning the phosphate-buffered saline was exchanged with a 30% sucrose PBS solution. The forebrain was separated from the cerebellum and mounted in the coronal plane with Tissue-Tek^® ^(Sakura Finetek Europe B.V., Zoeterwoude, Netherlands). The left non-ischemic hemispheres were marked with a fine needle prior to sectioning in order to be able to distinguish the two hemispheres during stereological tissue analysis. Each brain was cut into 60 μm thick coronal sections on a calibrated Leica cryostat (Leica, Germany) and sectioned exhaustively into 200-220 slices. All sections from one brain were divided in ten different wells filled with anti-freeze solution (30% glycerol, 30% ethylene-glycol, and phosphate-buffered saline) and stored at -20°C. The distance between the parallel sections from the same well was 600 μm.

### Immunohistochemistry

Brain sections from all animals were processed for immuno-labeling in the same run using a free floating tray system with 20 wells per tray [19]. Infarct volumes were estimated on neuronal nuclei (NeuN) stained tissue sections using a biotinylated primary antibody (1:1000; Cat. no. MAB377B; Chemicon International, Temecula, CA, USA). Neuronal precursor cell proliferation was visualized in a BrdU (1:200; Cat. no. 347580; Becton Dickinson, San José, CA, USA) and ED-1 (1:4000; Cat. no. MAB1435; Chemicon International, Temecula, CA, USA) double stain.

The first step in staining for BrdU was incubation in 50% formamide in 50% 2 × standard sodium citrate (2 × SSC) (0.3 mol/L NaCl and 0.03 mol/L sodium citrate) buffer at 65°C for 2 hours, followed by 30 minutes in 2N HCl at 37°C and a neutralizing step in 0.1 M boric acid (pH 8.5) for 10 minutes at room temperature. From here on all stains were treated uniformly. Endogenous peroxidase activity was blocked with 2% H_2_O_2 _in phosphate-buffered saline for 20 minutes. The tissue was incubated in 5% normal swine serum in 1% bovine serum albumin in phosphate-buffered saline + 0.3% Triton X for 30 minutes at room temperature before overnight incubation with one of the diluted primary antibodies in 1% bovine serum albumin in phosphate-buffered saline + 0.3% Triton X at 4°C.

The brain sections were incubated for 1 hour at room temperature with the biotinylated donkey-anti-mouse antibody (F(ab)_2_) dilution (Cat. no. 715-066-150; Jackson ImmunoResearch Laboratories INC., West Grove, PA, USA) in blocking buffer (1% bovine serum albumin in phosphate-buffered saline+0.3%Triton X). This procedure did not include the NeuN stain where the primary antibody was biotinylated. The sections were finally incubated with an avidin-biotin-peroxidase complex kit (Cat. no. PK-6100, Vector Laboratories, Burlingame, CA, USA) for 1 hour at room temperature before the development with nickel-enhanced DAB or NovaRed^® ^(Cat. no. SK-4100 and SK-4800, Vector Laboratories, Burlingame, CA, USA).

Brain sections were mounted on SuperFrost^® ^Plus slides (Menzel GmbH + Co KG, Braunschweig, Germany), air-dried and cover-slipped with Pertex^® ^(Bie & Berntsen, Rødovre, Denmark) using thin cover glasses.

### 2D nucleator

**Figure 3:**
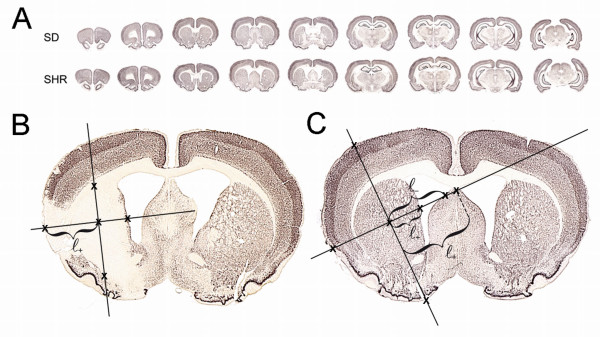
**Application of the 2D nucleator on NeuN-stained tissue sections**. **A **shows representative coronal NeuN-stained brain sections developed with nickel-enhanced DAB from outbred male sham SDs (first row) and SHRs (second row). Note the larger ventricular system and the flatter appearance of the brain on the coronal sections of SHRs. **B **and **C **are larger images of one of the NeuN stains from an animal with and without ischemic stroke. The cross-sectional area of the infarct (**B**) or the hemisphere (**C**) is estimated with the 2D nucleator [21,23]. Our estimates were based on computer-assisted measurements using CAST^® ^software (Visiopharm A/S, Hørsholm, Denmark). After marking the center in the region of interest, the computer generated four random, right-angled, isotropic directions. The intersection lengths (ℓ_+_) from the center point to the boundary were measured and the cross sectional area was estimated with the equation: . Cross sections of more complex structures included several intercepts from the center point to the boundary (**C**). ℓ^2 ^was calculated by adding the squared lengths with alternating opposite signs (ℓ_+ _or ℓ_-_). The intersection point most distant from the centre was always positive. Finally, the volume of the brain structure or infarct could be estimated using the Cavalieri principle, since: (1) the coronal slices were parallel; (2) the distance between the slices was known (i.e. 600 μm); and (3) the first coronal slice hitting the structure of interest was random. **NeuN**, neuronal nuclei; **SDs**, Sprague-Dawley rats; **SHRs**, spontaneously hypertensive rats.

A 2D area estimator and the Cavalieri principle were combined for the unbiased volume estimation of the brain hemispheres, non-neocortical structures, striatum, and infarct volumes [20-22]. An Olympus SZ-11 stereomicroscope (Olympus, Japan) equipped with a ColorView digital camera (Olympus Soft Imaging Solutions GmbH, Münster, Germany) was connected to a PC. The cross sectional areas were estimated unbiased with the 2D nucleator on NeuN stained coronal brain sections using CAST^® ^software (Visiopharm A/S, Hørsholm, Denmark). The centre of the region of interest was marked manually with the computer mouse. The computer generated a predetermined number of two perpendicular test lines with systematic random isotropic directions (Figure 3B and 3C). The length of the test lines (ℓ) from the manually marked center to the boundary was automatically measured, and the cross sectional area estimated by the computer as [21,23]:(1)

In the event that irregular boundaries were intersected more than once (Figure 3C), ℓ^2 ^was calculated by adding the squared lengths from the boundary centre point to the intersection points. The ℓ^2 ^had alternating opposite signs beginning with the intersection point most distant from the origin (ℓ_+_).

The volume of the structure of interest (e.g. hemisphere, striatum or infarct) was estimated using the Cavalieri principle based on the summed cross sectional area (*a *) estimates from the parallel coronal NeuN stained sections with a known distance (t) of 600 μm [20,22].(2)

The correct use of the Cavalieri principle for volume estimation requires that: (1) all sections of the object of interest are parallel; (2) the distance between the sections is constant and known; and (3) the position of the first coronal slice hitting the structure of interest is random [24].

### Optical fractionator

**Figure 4:**
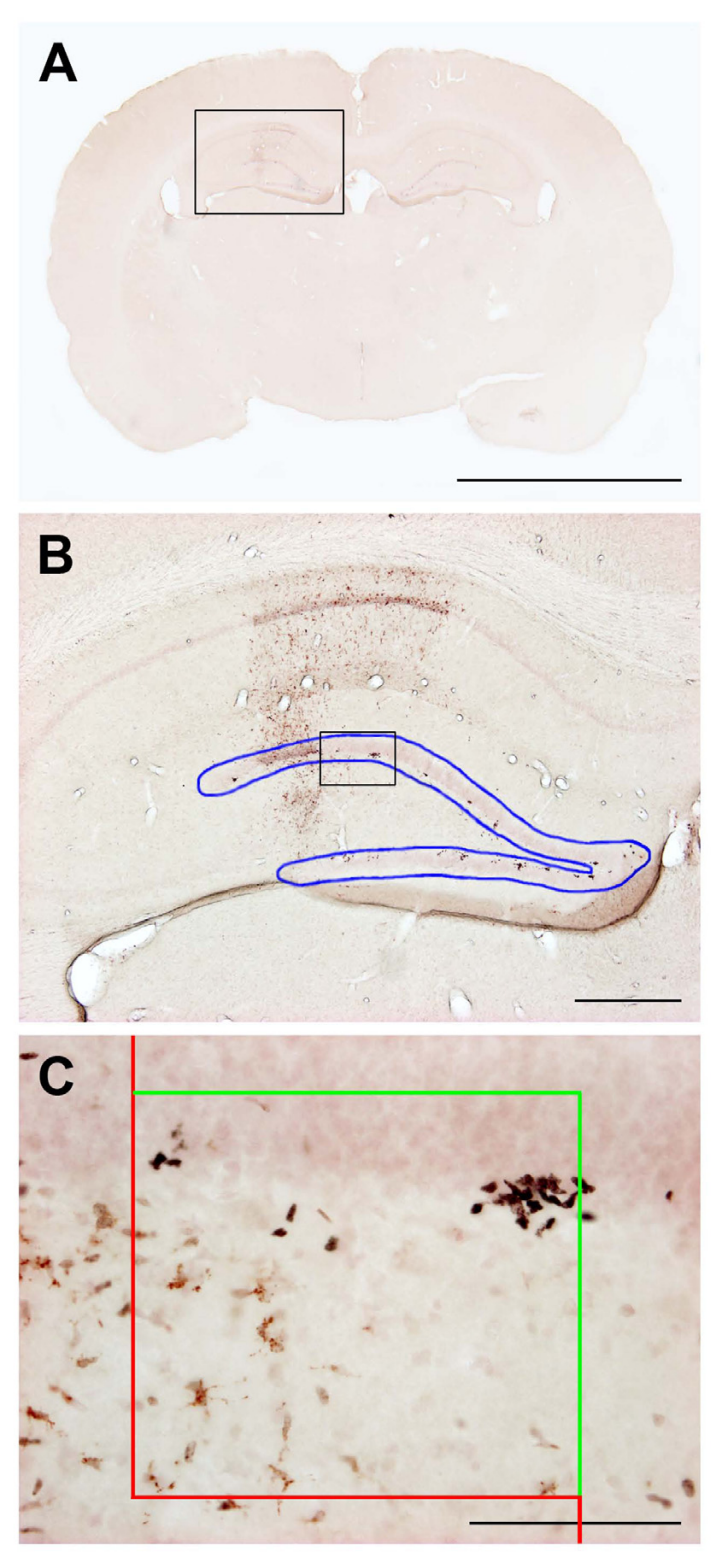
**The practical use of the optical fractionator**. The optical fractionator design was applied on 7-9 coronal sections covering the whole dentate gyrus. The sections were double-stained for BrdU and ED-1, mounted on glass slides, and cover-slipped with Pertex^® ^(Bie & Berntsen, Rødovre, Denmark) and thin cover glasses. CAST^® ^software (Visiopharm A/S, Hørsholm, Denmark) was used for the computer assisted counting. **A **shows an example of one of the coronal sections used for the counting of BrdU^+ ^cells. First, the SGZ of the DG was delineated with a 4× objective (**B**). A defined area sampling fraction (asf) within the delineated area was used for the counting procedure applying the principle of systematic unbiased random sampling. The asf was determined based on test counts where 150-200 BrdU^+ ^cells in each hemisphere were counted. The CAST^® ^software generated an unbiased counting frame as shown in **C**. BrdU^+ ^cells within the frame or intersecting the green inclusion lines were counted using a 40× objective. All cells intersecting or touching the red exclusion lines were not counted. The scale bars represent 5 mm (**A**), 500 μm (**B**), and 100 μm (**C**), respectively. **asf**, area sampling fraction; **BrdU**, 5-bromo-2'-deoxy-uridine; **DG**, dentate gyrus; **SGZ**, subgranular zone.

We counted the BrdU labeled cells in the DG of the hippocampus using the optical fractionator design to obtain an unbiased estimate of the number of proliferating NPCs in the two rat strains after tMCAo and sham. An Olympus BX50 light microscope (Olympus, Japan) equipped with a motorized specimen stage, a microcator (Heidenhain, Germany) and a 3-CCD video camera interfaced to a PC via a frame grabber was used for the stereologic counting. First, the DG was delineated with a 4× objective (Figure 4A). The CAST^® ^system (Visiopharm A/S, Hørsholm, Denmark) generated an unbiased counting frame with a 40× objective within the delineated DG area (Figure 4C). We only counted BrdU^+ ^cells within the counting frame or intersecting the inclusion lines. The unbiased estimated cell number was calculated with the equation below [25,26]:(3)

The section sample fraction (*ssf *) was 0.1 since we sampled from 1 out of 10 sections. The area sampling fraction (*asf *) was set to 0.25 or 25% based on preliminary counts where we aimed at counting 150 - 200 BrdU^+ ^cells in seven to nine different coronal sections per DG. The height sampling fraction (*hsf *) was 1, since the whole section thickness of 60 μm was used. Σ *Q*^- ^was the total number of counted BrdU^+ ^cells per DG. The counting procedure was preceded by an analysis of the z-axis distribution of BrdU^+ ^cells. The sections from both strains had a similar height and z-axis distribution.

### Statistics

All data were analyzed using Stata Intercooled 8.2 software (StataCorp, College Station, TX, USA). Infarct volumes were tested with Student's t test. One-way ANOVA was used for the between-group comparisons. P < 0.05 was considered statistically significant.

## Results

### Drop outs

The permanent ligation of the pterygopalatine artery just distal to the internal carotid artery bifurcation (Figure 2A &2B) is technically a demanding step in the intraluminal tMCAo model and two SD rats were excluded prior to randomization due to damage of the intracranial part of the internal carotid artery because this could have compromised a proper reperfusion phase. Two SD rats, one from either group, died within the first 24 hours after surgery. Brain autopsy showed no signs of intracranial hemorrhage in any of the two animals. Originally, thirteen animals were enrolled into the SHR ischemia group. However, two rats were in a bad clinical condition and were therefore sacrificed prematurely for ethical reasons. Autopsy revealed subarachnoid hemorrhage in both animals. Unexpectedly, one animal from the SHR sham group had histological changes consistent with a small cortical infarct on the ipsilateral (right) side. The latter animal was excluded from the stereological quantification of the BrdU incorporation in the DG.

### Physiological parameters

As expected the mean middle arterial blood pressure differed significantly between the anesthetized outbred SDs and SHRs. In SHRs it was generally 40 - 50 mmHg above that seen in SDs (Additional file 2). We found minor differences between the four groups in terms of heart rate, rectal temperature, pH, pCO_2_, pO_2_, hemoglobin, and glucose. However, such differences are typical in statistical analysis of small sample sizes and do not have any physiological importance.

The pCO_2 _was typically higher in the first arterial blood samples obtained before sham or tMCAo, and they tended to decrease in the second and third measurements due to a reduction in anesthesia depth compared with the initial measurements within 10 to 15 minutes anesthesia induction (Figure 2C).

As presented in Additional file 2 the average bodyweight loss was 12-15% from day 1 to day 8. This weight loss happened regardless whether the rats belonged to the SD or SHR strain and whether the animals were subjected to tMCAo or sham surgery. The most pronounced weight loss occurred during the first four days (Additional file 3). Thereafter, the mean bodyweight tended to stabilize or even slightly increase. Varying degrees of jaw and neck muscle ischemia within the right external carotid artery territory seem to be responsible for impaired mastication and swallowing which led to the striking postoperative weight loss [27,28].

### Volumes of brain structures

**Figure 5:**
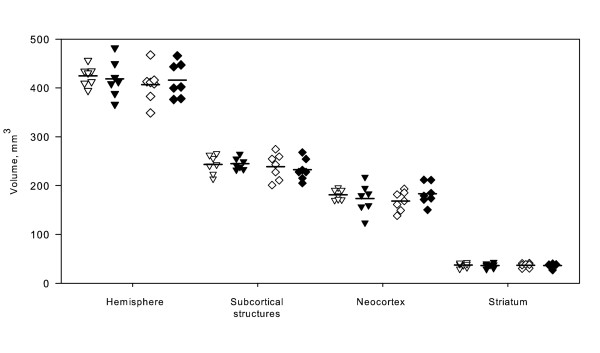
**Brain structure volume estimates**. Estimated volumes using the 2D nucleator and the Cavalieri principle are illustrated for hemispheres, subcortical structures, neocortices, and striatum. The presented volumes were estimated on NeuN-stained tissue sections on both sides in all sham animals and the mean volumes are summarized in Table 1. SD sham volumes are represented with "unfilled and filled triangles" for the left and right hemisphere, respectively. SHR sham volumes were likewise represented with "unfilled and filled diamonds". Mean volumes are marked with horizontal lines. **NeuN**, neuronal nuclei; **SD**, Sprague-Dawley rat; **SHR**, spontaneously hypertensive rat; **tMCAo**, transient middle cerebral artery occlusion.

Differences in brain structure volumes between the SD and SHR strain due to the larger ventricular system of SHRs compared with normotensive rat strains (Figure 3A) were estimated as volumes of hemispheres, subcortical structures, and striatum in all sham animals using the 2D nucleator and the Cavalieri principle (Figure 5 and Table 1). The volume of the neocortex was determined as the difference between the hemisphere and subcortical structure volumes. We defined the boundary of the neocortex from the rhinal fissure to the interhemispherical fissure, whereas the piriform cortex was considered a non-neocortical structure [29].

**Table 1:** Brain structure volume estimates.

Group	SDs left side (n = 7)	SDs right side (n = 7)	SHRs left side (n = 7)	SHRs right side (n = 7)
Hemisphere volume (mm^3^)	424.6 ± 20.4	418.4 ± 38.2	406.7 ± 36.0	415.9 ± 35.8
Subcortical volume (mm^3^)	243.4 ± 19.5	244.8 ± 12.0	238.6 ± 26.8	232.8 ± 21.7
Neocortical volume (mm^3^)	181.2 ± 10.9	173.5 ± 30.1	168.1 ± 20.1	183.1 ± 22.1
Striatal volume (mm^3^)	37.2 ± 4.9	36.5 ± 5.0	36.5 ± 4.6	36.2 ± 4.4

Mean volumes ± SD are summarized. SDs, Sprague-Dawley rats; SHR, spontaneously hypertensive rats.

The sham animals from both strains had a comparable mean bodyweight and were not affected by tissue loss due to tMCAo. We found no differences between the SHR and the SD strain. The mean hemisphere volume was estimated to 420 mm^3 ^where the subcortical area occupied 240 mm^3 ^and the neocortex 180 mm^3^. The striatum had an estimated volume of 37 mm^3^.

### Infarct volumes and stroke pattern

**Figure 6:**
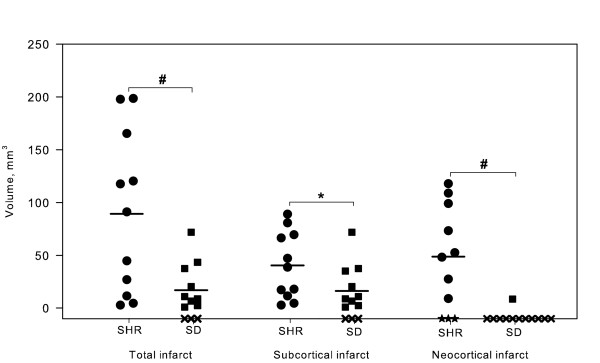
**Infarct volume estimates**. All eleven SHRs had infarct changes in the right hemisphere after 90 minutes of tMCAo, but three out of 13 SDs had no histological signs of infarct on the NeuN-stain. The infarct volume differed significantly between the two strains with a mean of 89.2 mm^3 ^for the SHRs and 16.9 mm^3 ^for the SDs (p < 0.005). The subcortical portion of the total infarct was 40.4 mm^3 ^for the SHRs compared with 16.2 mm^3 ^for the SDs (p < 0.044). The mean neocortical infarct volume was determined as the difference between the total infarct volume and the subcortical portion of the infarct. In eight out of eleven SHRs, we found neocortical involvement in the infarct as opposed to one animal out of 13 SDs. This reflects our general experience that SDs hardly get neocortical infarcts after 90 minutes of tMCAo. SDs are marked with "black squares", whereas "black circles" represent SHRs. Animals which did not have infarcts were marked with "black crosses" if they belonged to the SD strain or "black stars" if it were SHRs. Mean infarct volumes are marked with horizontal lines. * indicates p < 0.05, whereas p < 0.01 are marked with #. **NeuN**, neuronal nuclei; **SDs**, Sprague-Dawley rats; **SHRs**, spontaneously hypertensive rats.

The estimated total mean infarct volumes differed significantly and were 89.2 mm^3^± 76.1 mm^3 ^for the SHRs compared with 16.9 mm^3 ^± 22.7 mm^3 ^for the SDs (p < 0.005) (Figure 6). The mean subcortical part of the infarcts comprised 40.3 mm^3^± 31.8 mm^3 ^in the SHRs and 16.2 mm^3^± 22.0 mm^3 ^in the SDs (p < 0.044) (Figure 6). In SHRs the neocortical infarct had a mean size of 48.7 mm^3^± 45.5 mm^3^. All eleven SHRs had ischemic damage within the right hemisphere following 90 minutes of tMCAo. Eight SHRs had ischemia in both neocortex and the subcortical structures, whereas three animals only had subcortical infarcts. Nine out of twelve SDs showed ischemic damage on the NeuN stain. Except for one animal, all infarcts were restricted to the subcortical structures in SDs. However, three SDs showed no histological signs of neuronal loss or microglia activation at all.

**Figure 7:**
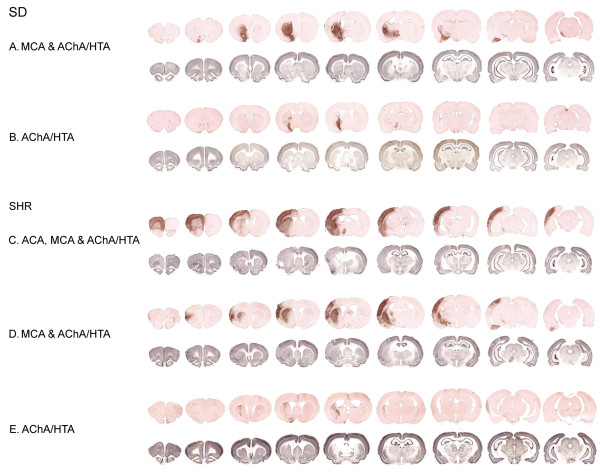
**Infarct distribution in SDs and SHRs**. Representative examples are shown of the infarct distribution after 90 minutes of tMCAo in the SD and SHR strains using NeuN and ED-1 immunohistochemistry. The ED-1 (brownish red rows) was visualized with NovaRed^®^, whereas NeuN stain was developed with nickel-enhanced DAB (grey rows). **A **and **B **depict the infarct pattern we found in SDs. Usually, the infarcts were restricted to the subcortical structures in SDs encompassing both the MCA and AChA/HTA territories (4 SDs) (**A**) or the AChA/HTA territories alone (5 SDs) (**B**) Three of the tMCAo SDs had no histological signs of neuronal loss on the NeuN stain. **C**, **D **and **E **illustrate the infarct distribution in SHRs. In most of the SHRs subjected to ischemia the infarcted area included the neocortex (8 out of 11 SHRs). **C **shows an example of ischemia affecting the ACA, MCA and AChA/HTA (2 SHRs). The majority of animals had ischemia in the MCA and AChA/HTA territories (8 SHRs) (**D**). Only one SHR had a small ischemic lesion solely affecting the AChA/HTA territories (**E**). **ACA**, anterior cerebral artery; **AChA**, anterior choroidal artery; **DAB**, 3,3'-diaminobenzidine; **HTA**, hypothalamic artery; **MCA**, middle cerebral artery; **NeuN**, neuronal nuclei; **SDs**, Sprague-Dawley rats; **SHRs**, spontaneously hypertensive rats; **tMCAo**, transient middle cerebral artery occlusion.

Representative infarct distributions in the two strains are illustrated in Figure 7. We found two infarct patterns in the SD strain involving the territories of the anterior choroidal artery and the hypothalamic artery or a combination of the anterior choroidal artery/hypothalamic artery and the middle cerebral artery territories. Middle cerebral artery infarction affected the fronto-parietal neocortex and most of the striatum, notably its rostro-lateral part. Involvement of the anterior choroidal artery/hypothalamic artery was defined as infarction of the caudo-medial part of the striatum encompassing the internal capsule and parts of the hypothalamus. In addition, we observed involvement of the anterior cerebral artery in two of the SHRs where the frontal cortex had undergone ischemic degeneration.

### Neuronal precursor cell proliferation

**Figure 8:**
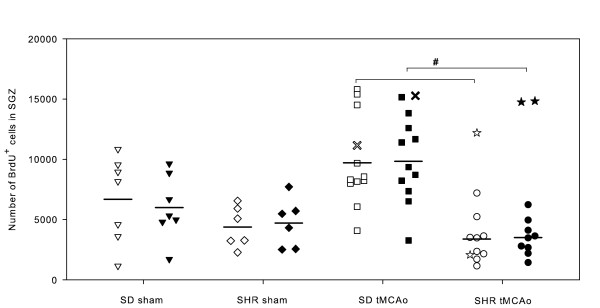
**Estimated numbers of BrdU^+ ^cells in the SGZ of SDs and SHRs**. We found a very large spread in the number of BrdU^+ ^cells within both strains and in each of the four groups. Even among sham animals, the number of BrdU^+ ^cells in the SGZ could vary ten-fold. We were not able to demonstrate a statistically significant increase in the BrdU incorporation one week after tMCAo. Further, we found no difference between the sham groups from both strains. Interestingly, we saw a marked increase in BrdU uptake in the ipsilateral DG in three animals (2 SHRs and 1 SD) where the ischemic damage involved the hippocampus (Figure 9). We found a statistically significant difference in the response to ischemia between the two strains if these three animals were excluded from the statistical analyses of the BrdU data. This could indicate that SDs have a higher NPC proliferation than SHRs after remote ischemia. SD sham and SD tMCAo are marked with "triangles" and "squares", whereas SHR sham and SHR tMCAo are represented with "diamonds" and "circles". Note that the filled symbols represent right hemispheres, whereas the unfilled symbols represent left hemispheres. Animals with an ischemically injured hippocampus that were excluded from the statistical analyses were marked with "crosses" if they belonged to the SD strain or "stars" if they were SHRs. Mean BrdU^+ ^cell numbers are marked with horizontal lines. * indicates p < 0.05, whereas p < 0.01 are marked with #. **BrdU**, 5-bromo-2'-deoxy-uridine; **DG**, dentate gyrus; **SDs**, Sprague-Dawley rats; **SGZ**, subgranular zone; **SHRs**, spontaneously hypertensive rats; **tMCAo**, transient middle cerebral artery occlusion.

The post-stroke BrdU incorporation on day 4 to 7 was investigated in the subgranular zone of the dentate gyrus (DG) using the optical fractionator principle (Figure 4). The sham animals from both strains had a comparable number of BrdU^+ ^cells, although there was a considerable, up to ten-fold spread in the estimated numbers (Figure 8). The mean number of BrdU^+ ^cells in the subgranular zone in both hemispheres rose in SDs after ischemia, but did not differ significantly from the mean values in sham animals. We found no difference between the sham and tMCAo SHRs, but the mean BrdU^+ ^cell number was even lower in the animals subjected to ischemia. In all four groups the number of BrdU^+ ^cells was symmetrically distributed in both hemispheres.

**Figure 9:**
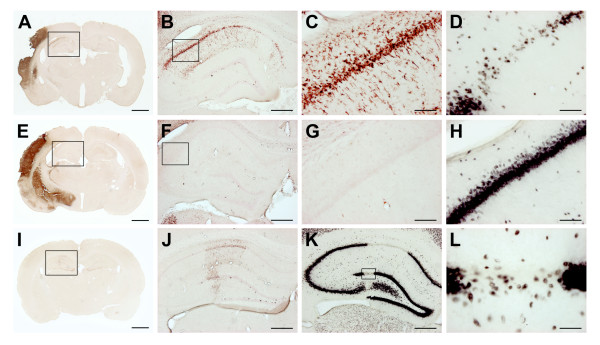
**Examples of BrdU incorporation in the SGZ in rats with different infarct distribution**. The two first rows (**A**-**D **and **E**-**H**) are BrdU/ED-1 and NeuN-stained sections from two SHRs with a similar infarct size (120.3 mm^3 ^vs. 117.6 mm^3^) but with a different infarct distribution. In the last row (**I**-**L**), sections are shown from a SD rat with a very small infarct (0.95 mm^3^) solely confined to the right DG. The magnitude of the BrdU uptake in the right DG was similar in the SHR visualized in **A**-**D **and the SD shown in **I**-**L **(14840 BrdU^+ ^cells vs. 15280 BrdU^+ ^cells), whereas the SHR represented in **E**-**H **had a much lower BrdU uptake (2800 BrdU^+ ^cells). **A**-**C**, **E**-**G**, and **I**-**J **are BrdU/ED-1 double stains. The boxed area on the coronal sections in **A**, **E**, and **I **are shown in **B**, **F **and **J**-**K**, whereas the boxed area in **B **and **F **are depicted in **C**-**D **and **G**-**H**. **D**, **H**, and **K**-**L **are NeuN stains that correspond to the BrdU/ED-1 double stain in **C**, **G**, and **J**. Note the infiltration of ED-1^+^-activated microglia in **B**-**C**, and **J **compared with **F**-**G**. Correspondingly, neuronal loss was found in **D **and **K**-**L**, but not in **H**. The presence of activated microglia in the microenvironment around the DG, however, did not seem to compromise the BrdU uptake in the SGZ. The scale bars represent 1 mm (**A**, **E**, **I**), 500 μm (**B**, **F**, **J**, **K**), 100 μm (**C**, **D**, **G**, **H**), and 50 μm (**L**) respectively. **BrdU**, 5-bromo-2'-deoxy-uridine; **DG**, dentate gyrus; **NeuN**, neuronal nuclei; **SDs**, Sprague-Dawley rats; **SGZ**, subgranular zone; **SHRs**, spontaneously hypertensive rats.

It was not possible to correlate the BrdU uptake in the subgranular zone with infarct volume in the two ischemia groups. However, in one SD and two SHRs subjected to tMCAo, the ischemic injury involved the hippocampus (Figure 9). In these three animals, the total number of BrdU^+ ^cells in the subgranular zone rose considerably in the ipsilateral DG, which made the animals to deviators in their respective groups (Figure 8). The presence of ED-1^+^-activated microglia around the DG did not seem to compromise NPC proliferation. The magnitude of the BrdU incorporation in the contralateral non-ischemic subgranular zone varied in the three deviators. In two (one SD and one SHR), the contralateral BrdU uptake was high, whereas in the third (SHR) the BrdU incorporation was quiet low.

We found a significant difference in the post-ischemic BrdU uptake between the two strains if the three deviators were excluded from the statistical analyses (Figure 8). This difference could indicate that the NPC proliferation response after remote ischemic injury is different in the two strains.

## Discussion

The most intriguing findings in this study were our observations that: (1) NPC proliferation in the subgranular zone did not increase one week after remote transient ischemia in SHRs and SDs; (2) direct ischemic injury in the hippocampus stimulated BrdU uptake in the subgranular zone profoundly; and (3) presence of ED-1^+ ^activated microglia in the DG did not compromise NPC proliferation. We linked the increased BrdU uptake to infarction in the anterior choroidal artery territory that, with some variation, includes the DG [29,30].

### Stereological tissue analysis

Brain structure volume, infarct volume, and BrdU^+ ^counts in DG were estimated using design-based stereological tools; an approach that requires some planning. The advantage of this approach is that information about three-dimensional, microscopic structures may be obtained from tissue sections, although every step in the tissue processing from fixation to mounting affects the quantitative information obtained [25]. Especially the fixation and embedding procedures contribute considerably to tissue deformation [25]. Unfortunately, many investigators use paraffin-embedded brain tissue sections where the deformation is pronounced and unpredictable in the x-, y-, and z-axes. We decided to use cryostat sections in the present study because freezing and vibratome sections tend to be stable in the x and y axes. This enabled us to estimate the surface area of the region of interest using the 2D nucleator without correction for x-, and y-axis shrinkage.

In 2D stereology the estimated parameter (area) can be directly observed on the slide through a microscope. The design-based stereological methods used for estimation of surface area can, for practical reasons, be divided into two different approaches: (1) procedures that involve simple point counting; and (2) tools that require measurements (e.g. the 2D nucleator) [23]. The 2D nucleator is a member of a larger nucleator family for size estimation in the *n *-dimensional space introduced by Gundersen [21]. Its practical use has been greatly improved by the introduction of computer-assisted test systems. The use of sterological software like CAST^® ^(Visiopharm, Hørsholm, Denmark) enabled the generation of perpendicular test lines with isotropic directions by a simple click on the computer mouse. After the boundary of the region of interest (e.g. striatum or infarct) had been manually marked on each test line, the computer calculated the surface area according to equation 1.

We estimated the number of BrdU^+ ^cells in the hippocampal DG as a measure of NPC proliferation using the optical fractionator design [25,26]. It was necessary to check the section thickness at the position of a sufficiently large number of BrdU^+ ^cells (e.g. 250 to 300 BrdU^+ ^cells in the subgranular zone of the DG) due to non-uniform deformation in cryostat sections in the direction of the z-axis [25]. The latter procedure is called a z-axis distribution. The z-axis deformation may be down to less than 1/3 of the original section thickness in cryosections. In the present study the mean height of the tissue sections was around 16 μm. The considerable shrinkage forced us to perform the counting procedure through the whole section height without guard zones in the z-axis. Therefore our NPC data are not unbiased as surface irregularities introduced by cutting were not corrected. Some of the z-axis shrinkage in cryostat sections can be counterbalanced with the use of aqueous mounting media directly on wet-mounted tissue sections. However, immuno-stained tissue sections mounted with aqueous mounting media have a shorter lifetime. It is our experience that optimization of the immunohistochemistry protocol on thick tissue sections (e.g. 60 μm or above) is required due to penetration problems with the primary antibodies. Further introduction to the application of stereology in neuroscience can be found in Dorph-Petersen et al. [25], and Evans et al. [31].

### Intraluminal tMCAo in rats

Since the mid 80s, the intraluminal filament tMCAo model introduced by Koizumi et al. [32] and Longa et al. [17] has gained popularity for a number of reasons. First, it is a feasible surgical technique; second, it adopts a closed skull approach; and third, it affords the possibility of achieving transient or permanent focal brain ischemia [33]. Since its introduction, a variety of pitfalls and complications in using the model have regularly been reported [27,34-39]. These reports have primarily addressed the difficulties of infarct reproducibility and reliability in the intraluminal tMCAo model and pointed to a variety of method-specific and method-unspecific parameters that shape the outcome after endovascular tMCAo [18,33,39]. The use of laser-doppler flow measurements of the cerebral blood flow during the endovascular occlusion is the preferred method for monitoring the completeness of middle cerebral artery blockage and controlling for adverse effects like intracerebral hemorrhage and subarachnoid hemorrhage [37,40]. Still, a recent report by Prieto et al. [41] indicates that there are considerable strain differences in the laser-doppler flow response measured after tMCAo.

In the present study, the ischemic damage was determined histologically using NeuN and ED-1 as markers (Figure 7). ED-1 is a lysosomal macrophage antigen that is expressed in microglia upon activation as seen following tMCAo [42]. Activated microglia is a very sensitive tissue injury marker [43-45]. The use of free-floating immunohistochemistry increases the workload considerably compared with conventional techniques like the 2,3,5-triphenyltetrazolium chloride stain [46]. However, relatively small infarcts are easily missed if the brain is not sectioned exhaustively, and immunohistochemical approaches offer the advantage of multiple antigen labeling.

### Infarct size and distribution after endovascular tMCAo

The SHR strain exhibits an increased susceptibility to permanent [8,9,11-13] and transient [10] brain ischemia compared with other normotensive rat strains. We found the same strain characteristics in this study, although we observed a large spread in infarct volume which could be due to the use of uncoated filaments (microloaders) as proposed by Laing et al. [34] and Schmid-Elsaesser et al. [37]. Barone et al. [10] have thoroughly reviewed the evidence for the increased ischemic sensitivity of the SHR strain and outlined two key hemodynamic factors of importance: (1) high vascular resistance; and (2) compromised blood flow through collateral vessels.

Vascularization of the non-neocortical structures in the rat is extensive and complex [47]. The anterior choroidal artery and the hypothalamic artery are two small arteries arising from the intracranial part of the internal carotid artery just proximal to the origin of the middle cerebral artery [30]. Blockage of the middle cerebral artery by an endovascular thread therefore involves the risk of infarction in the territories supplied by the anterior choroidal artery and the hypothalamic artery depending on the diameter of the filament [29,30]. Besides extending the volume of the infarct, infarction of these two arteries may also entail spontaneous postischemic hyperthermia which aggravates the ischemic brain injury [29,48-50].

The infarct distribution in the present study was defined according to He et al. [29]. We found involvement of the right anterior choroidal artery/hypothalamic artery territories in all animals from both strains as shown in Figure 7. We found no clinical evidence of hypothalamic artery infarction. However, postischemic hyperthermia could not be completely ruled out since the postsurgery body temperature was not monitored. He et al. [30] reported that hippocampal ischemia occurred in 17% of female Wistar tMCAo rats after an isolated anterior choroidal artery infarct and in 36% of the animals after a combined anterior choroidal artery/middle cerebral artery infarct. In our study, three out of 23 (13%) tMCAo animals had ischemic neuronal degeneration in the ipsilateral hippocampus (Figure 9).

We observed an unexpected, isolated neocortical infarction on the ipsilateral side after a sham procedure in one SHR. This incident could be due to secondary thromboembolism formation following common carotid artery clamping or filament-induced endothelial injury. The SHR strain may thus be more vulnerable to embolus formation due to the impaired collateral cerebral circulation.

### NPC proliferation in the dentate gyrus after stroke

We administered BrdU (50 mg/kg bodyweight) from Day 4 to 7 after surgery to mark the proliferating NPC in the subgranular zone of the DG (Figure 2D). This administration protocol was based on previous reports where NPC proliferation was found to peak 4-10 days after injury-induced neurogenesis [51-53]. In addition, it is proposed that mitotically active cells do not differentiate into neurons at early time points after injury [53].

It remains controversial whether direct injury to the hippocampus is necessary to induce an increased NPC proliferation. In models examining poststroke hippocampal neurogenesis after transient forebrain ischemia [52,54,55], it was observed that neuronal hippocampal injury was reproducible because the CA1 region is particularly eligible to ischemia in these models. Likewise, electroconvulsive therapy that affects the whole brain was found to stimulate hippocampal neurogenesis [56]. In a recent report by Kluska et al. [57], anterior photothrombotic infarction stimulated BrdU uptake in the DG 4 weeks after injury but not after 10 weeks, whereas posterior lesions close to the hippocampus increased BrdU incorporation at both time points. Ernst et al. [53] reported that insertion of electrodes into cortical layer 6 above the hippocampus did not lead to increased NPC proliferation. However, direct and discrete trauma to the DG significantly increased mitotic activity in the subgranular zone and hilus. The latter situation resembles the situation shown in Figure 9I-9L where the animal had a very small infarct restricted to the ipsilateral DG.

Several studies have shown that focal brain ischemia without involvement of hippocampal tissue increased poststroke neurogenesis in rats [51,58-60]. In general, it is difficult to compare the mentioned studies due to differences in animal models, BrdU protocols, and counting procedures. Still, we found no similar clear response in this study. There was a symmetrical increase in the mean number of BrdU^+ ^cells in the SD strain after tMCAo, but it did not reach statistical significance. The mean BrdU^+ ^cell number was not much affected by the three SDs that sustained no ischemic injury following the tMCAo procedure. Exclusion of these animals does not change the overall conclusions stated here. Although, it cannot be ruled out that SDs with larger infarcts comprising both the caudoputamen and neocortex could have increased NPC proliferation in the subgranular zone of the DG.

We even saw an insignificant decrease in the mean number of BrdU^+ ^cells in the SHR strain after tMCAo, if the three animals with hippocampal ischemic injury were left out of the statistical analyses. This trend is surprising since Perfilieva et al. [14] showed that the SHR strain has a higher basal BrdU incorporation in the subgranular zone than the SDs. The age difference could account for part of the observed difference between the two strains. However, it was reported by Darsalia et al. [61] that in aged rats, BrdU incorporation increases in the ipsilateral granular cell layer after tMCAo. On the contrary, it is known that the hippocampal volume decreases with age in SHRs [62]. Furthermore, it is proposed that the regressive changes and astroglial reactions in the hippocampus of aged SHRs could mimic vascular dementia seen in humans [63]. Palmer et al. [64] have stressed the importance of the angiogenic niche in adult hippocampal neurogenesis. Taken together, our findings could indicate that poststroke NPC proliferation is compromised in SHRs compared with normotensive animals.

## Conclusions

In this study we found no evidence of an early increased NPC proliferation in the subgranular zone one week after transient focal brain ischemia in SDs and SHRs. Infarction within the anterior choroidal artery territory can induce ischemic neuronal death in the hippocampus and trigger increased BrdU incorporation in the DG. NPC proliferation in animals with hippocampal ischemia was not affected by the presence of ED-1^+ ^activated microglia.

As expected, transient thread occlusion of the middle cerebral artery produced a significantly larger stroke volume with neocortical involvement in SHRs. However, endovascular tMCAo leads to total or partial occlusion of the anterior choroidal and hypothalamic arteries in both SDs and SHRs. In addition, the advancement of the occluding thread beyond the middle cerebral artery origin induced anterior cerebral artery infarction in 2 SHRs.

## List of abbreviations

**ABC**: Avidin-Biotin-peroxidase Complex; **AChA**: Anterior Choroidal Artery; **BPM**: Beats Per Minute; **BrdU**: 5-bromo-2'-deoxy-uridine; **BSA**: Bovine Serum Albumin; **BW**: Body Weight; **CCA**: Common Carotid Artery; **DAB**: 3,3'-DiAminoBenzidine; **DG**: Dentate Gyrus; **ECA**: External Carotid Artery; **FA**: Femoral Artery; **Hb**: Hemoglobin; **HR**: Heart Rate; **HTA**: Hypothalamic Artery; **ICA**: Internal Carotid Artery; **IHC**: ImmunoHistoChemistry; **IM**: IntraMuscular; **IP**: IntraPeritoneal; **IV**: IntraVenous; **LA**: Lingual Artery; **MA**: Maxillary Artery; **MABP**: Mean Arterial Blood Pressure; **MCA**: Middle Cerebral Artery; **NeuN**: Neuronal Nuclei; **NPC**: Neuronal Precursor Cell; **N_2_O**: Nitrous Oxide; **NSS**: Normal Swine Serum; **OA**: Occipital Artery; **O_2_**: Oxygen; **PA**: Pterygopalatine Artery; **PBS**: Phosphate Buffered Saline; **PCA**: Posterior Cerebral Artery; **SAH**: SubArachnoid Hemorrhage; **SHRs**: Spontaneously Hypertensive Rats; **STA**: Superior Thyroid Artery; **SVZ**: SubVentricular Zone; **tMCAo**: transient Middle Cerebral Artery occlusion; **TX**: Triton X.

## Competing interests

All authors declare that they have no conflict of interests. JK was supported by grants from the Institute of Clinical Medicine, University Hospital of Aarhus. LCBR was supported by the European Union Biomed Project GRIPANNT 005320. The MIND Center is funded by the Lundbeck Foundation. This study was supported by the Alice Brenaa Memorial Foundation, the Hede Nielsen Family Foundation, the A.P. Møller Foundation for the Advancement of Medical Science, the Helga and Peter Korning Foundation, and the University of Aarhus Research Foundation.

## Authors' contributions

JK designed the study, performed all animal experiments and drug administration, did all tissue sectioning, staining, mounting and counting, analyzed data, and wrote the paper. MHL advised on the BrdU administration protocol and immunohistochemistry. JCS, AM, JF and SN helped draft the manuscript. JRN advised in the use of stereologic tools, helped in statistical analyses and data interpretation. JM and LCBR helped designing the study, provided lab facilities, helped to interpret data, and drafted the manuscript. All authors read and approved the final manuscript.

## Supplementary Material

Additional file 1 **Microloader dimensions**. The diameters of the rounded microloaders were measured with CAST^® ^software (Visiopharm A/S, Hørsholm, Denmark). Mean values ± SD are shown. One-way ANOVA with Bonferroni post hoc analysis was used for the comparison between the groups. * indicates p < 0.05. **SD**, Sprague-Dawley; **SHR**, spontaneously hypertensive rat; **tMCAo**, transient middle cerebral artery occlusion. Click here for file

Additional file 2 **Physiological parameters**. Physiological parameters monitored before, during and after intraluminal tMCAo in SDs and SHRs. Mean values are presented ± SD. One-way ANOVA with Bonferroni post hoc analysis was used for the between group comparisons. * indicates p < 0.05, whereas p < 0.01 is marked with #. **HR**, heart rate; **MABP**, middle arterial blood pressure; **Rectal Temp**., rectal temperature; **SD**, Sprague-Dawley; **SHR**, spontaneously hypertensive rat; **tMCAo**, transient middle cerebral artery occlusion. Click here for file

Additional file 3 **Post-surgery development in animal body weight**. All animals in the four groups were weighed daily (Figure 2D). There was a pronounced decrease in mean BW within the first four days after surgery in both strains. In general, the animals lost 12-15% of their preoperative weight. The weight curves tended to stabilize from Day 5 and on. Note that the SHRs had a lower mean BW although they were 6 weeks older than the SDs. SD sham and SD tMCAo are marked with "black triangles" and "black squares", whereas SHR sham and SHR tMCAo are represented with "black diamonds" and "black circles". **BW**, bodyweight; **SDs**, Sprague-Dawley rats; **SHRs**, spontaneously hypertensive rats; **tMCAo**, transient middle cerebral artery occlusion. Click here for file
