# Supplementary material for: Neuronal precursor cell proliferation in the hippocampus after transient cerebral ischemia: a comparative study of two rat strains using stereological tools
Source: Exp Transl Stroke Med. 2010 Apr 6;2:8. doi: 10.1186/2040-7378-2-8 (PMC2868803; doi:10.1186/2040-7378-2-8)
Supplement: Additional file 1 — Microloader dimensions. The diameters of the rounded microloaders were measured with CAST® software (Visiopharm A/S, Hørsholm, Denmark). Mean values ± SD are shown. One-way ANOVA with Bonferroni post hoc analysis was used for the comparison between the groups. * indicates p < 0.05. SD, Sprague-Dawley; SHR, spontaneously hypertensive rat; tMCAo, transient middle cerebral artery occlusion. [file 2040-7378-2-8-S1.PDF]

Group	SD, sham (n = 7)	SD, tMCAo (n = 12)	SHR, sham (n = 7)	SHR, tMCAo (n = 11)
Filament tip diameter (μm)	296.6 ± 15.9	274.7 ± 17.0*	275.3 ± 12.2	289.9 ± 16.1
Filament diameter, 1 mm (μm)	216.7 ± 19.3	211.4 ± 19.5	219.7 ± 18.7	222.0 ± 18.3
Filament diameter, 10 mm (μm)	224.2 ± 33.4	224.8 ± 27.1	246.9 ± 36.1	228.6 ± 30.0
Filament diameter, 20 mm (μm)	230.1 ± 40.2	234.2 ± 35.5	251.5 ± 41.9	232.6 ± 40.2
