# Supplementary material for: Neuronal precursor cell proliferation in the hippocampus after transient cerebral ischemia: a comparative study of two rat strains using stereological tools
Source: Exp Transl Stroke Med. 2010 Apr 6;2:8. doi: 10.1186/2040-7378-2-8 (PMC2868803; doi:10.1186/2040-7378-2-8)
Supplement: Additional file 2 — Physiological parameters. Physiological parameters monitored before, during and after intraluminal tMCAo in SDs and SHRs. Mean values are presented ± SD. One-way ANOVA with Bonferroni post hoc analysis was used for the between group comparisons. * indicates p < 0.05, whereas p < 0.01 is marked with #. HR, heart rate; MABP, middle arterial blood pressure; Rectal Temp., rectal temperature; SD, Sprague-Dawley; SHR, spontaneously hypertensive rat; tMCAo, transient middle cerebral artery occlusion. [file 2040-7378-2-8-S2.PDF]

Group	SD, sham (n = 7)	SD, tMCAo (n = 12)	SHR, sham (n = 7)	SHR, tMCAo (n = 11)
MABP, before (mmHg)	90.4 ± 11.4	95.3 ± 12.4	143.7 ± 29.1#	152.2 ± 14.7#
MABP, during (mmHg)	83.2 ± 3.4	90.9 ± 9.8	135.2 ± 28.0#	142.1 ± 13.9#
MABP, after (mmHg)	86.7 ± 5.8	90.4 ± 10.9	142.0 ± 19.6#	130.8 ± 30.5#
HR, before (BPM)	346 ± 14	350 ± 20	345 ± 38	354 ± 22
HR, during (BPM)	354 ± 19	370 ± 22	338 ± 30*	370 ± 22
HR, after (BPM)	342 ± 20	349 ± 25	331 ± 22	344 ± 27
Rectal Temp., before (ºC)	36.9 ± 0.4	37.2 ± 0.4	37.3 ± 0.6	37.5 ± 0.5
Rectal Temp., during (ºC)	37.5 ± 0.2*	37.6 ± 0.1	37.7 ± 0.1	37.7 ± 0.1
Rectal Temp., after (ºC)	37.1 ± 0.5	37.2 ± 0.4	37.3 ± 0.4	37.4 ± 0.3
pH, before	7.40 ± 0.03	7.40 ± 0.03	7.45 ± 0.05	7.44 ± 0.03
pH, during	7.41 ± 0.05	7.39 ± 0.04	7.43 ± 0.03	7.43 ± 0.02*
pH, after	7.39 ± 0.03	7.38 ± 0.04	7.42 ± 0.03*	7.41 ± 0.03
pCO_2, before (kPa)	6.46 ± 0.73	6.28 ± 0.48	5.62 ± 0.62*	5.82 ± 0.42
pCO_2, during (kPa)	5.87 ± 0.91	5.95 ± 0.83	5.34 ± 0.22	5.41 ± 0.36
pCO_2, after (kPa)	5.71 ± 0.38	6.03 ± 0.64*#	5.31 ± 0.34	5.32 ± 0.39
pO_2, before (kPa)	29.81 ± 2.55	27.14 ± 3.09	27.08 ± 3.41	28.53 ± 2.16
pO_2, during (kPa)	27.53 ± 2.32	23.52 ± 5.11	27.34 ± 2.27	26.27 ± 1.55
pO_2, after (kPa)	26.82 ± 2.97	23.80 ± 4.55*	28.42 ± 1.35	26.78 ± 1.72
Hemoglobin, before (mmol/L)	9.4 ± 0.3*	9.6 ± 0.4	9.9 ± 0.3	9.9 ± 0.3
Hemoglobin, during (mmol/L)	8.4 ± 0.6*	9.0 ± 0.7	8.6 ± 0.4	9.2 ± 0.4
Hemoglobin, after (mmol/L)	7.9 ± 0.4	8.6 ± 0.7	8.2 ± 0.6	8.5 ± 0.3
Glucose, before (mmol/L)	12.1 ± 1.7	13.8 ± 2.4	14.6 ± 1.5	14.4 ± 1.7
Glucose, during (mmol/L)	8.5 ± 0.8	9.7 ± 1.6	9.7 ± 2.5	11.6 ± 1.3*#
Glucose, after (mmol/L)	8.4 ± 1.4	9.4 ± 1.7	10.8 ± 2.3	10.3 ± 2.5
Bodyweight, Day 1 (Gram)	332 ± 10	345 ± 17*#	318 ± 9	330 ± 11
Bodyweight, Day 8 (Gram)	293 ± 18	302 ± 20*#	273 ± 9	282 ± 13
Weight loss (Gram)	39 ± 25	43 ± 19	45 ± 8	48 ± 9
Anesthesia (min)	162 ± 10	155 ± 10	163 ± 24	165 ± 26
