# Supplementary figures and images for: Neuronal precursor cell proliferation in the hippocampus after transient cerebral ischemia: a comparative study of two rat strains using stereological tools
Source: Exp Transl Stroke Med. 2010 Apr 6;2:8. doi: 10.1186/2040-7378-2-8 (PMC2868803; doi:10.1186/2040-7378-2-8)

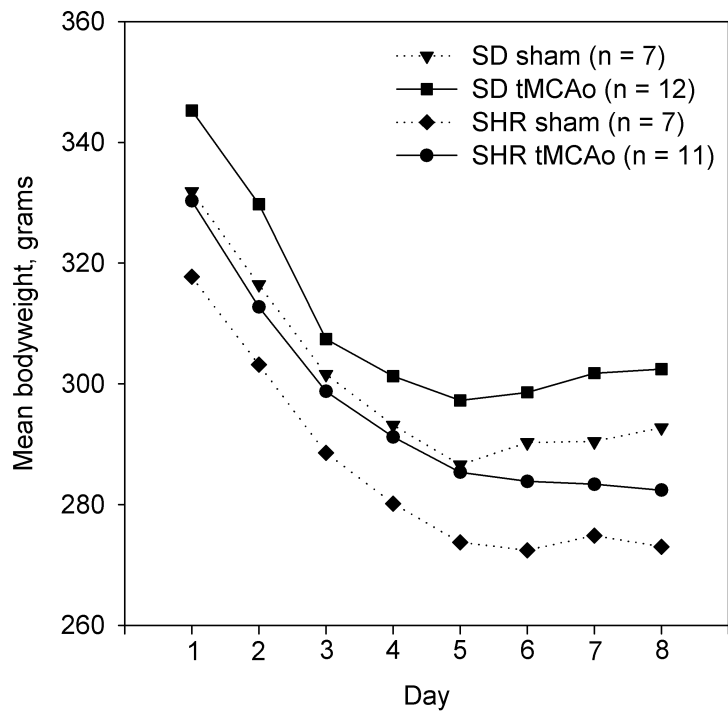

Supplement: Additional file 3 — Post-surgery development in animal body weight. All animals in the four groups were weighed daily (Figure 2D). There was a pronounced decrease in mean BW within the first four days after surgery in both strains. In general, the animals lost 12-15% of their preoperative weight. The weight curves tended to stabilize from Day 5 and on. Note that the SHRs had a lower mean BW although they were 6 weeks older than the SDs. SD sham and SD tMCAo are marked with "black triangles" and "black squares", whereas SHR sham and SHR tMCAo are represented with "black diamonds" and "black circles". BW, bodyweight; SDs, Sprague-Dawley rats; SHRs, spontaneously hypertensive rats; tMCAo, transient middle cerebral artery occlusion. [file 2040-7378-2-8-S3.PDF]
